# Correlation of psychooncological distress- screening and quality of life assessment in neurosurgical patients

**DOI:** 10.18632/oncotarget.22802

**Published:** 2017-11-30

**Authors:** Kira Hoffmann, Marcel Kamp, Hans-Jakob Steiger, Michael Sabel, Marion Rapp

**Affiliations:** ^1^ Department of Neurosurgery, Medical Faculty, Heinrich-Heine-University, Duesseldorf, Germany

**Keywords:** glioma, HADS, distress thermometer, Po-BADO, screening

## Abstract

**Background:**

Cerebral tumors are associated with high rates of anxiety, depression and reduced health related quality of life. But still psychooncological screening instruments are not implemented in the daily routine of neurosurgical departments. In contrast the EORTC QLQ-C30/ EORTC QLQ- BN20 questionnaire is often used to evaluate quality of life in the framework of clinical studies. We were therefore interested, if conspicuous distress screening results are also reflected by HRQOL assessment.

**Patients and Methods:**

Patients who were electively admitted for surgery of intracranial lesions were screened for their psychooncological distress using two self-assessment instruments (Hospital Anxiety and Depression Scale (HADS) and Distress Thermometer (DT)) and one external assessment questionnaire (Psychooncological base documentation (PO-Bado). Results were correlated with three subscales of the EORTC-QLQ-C30 and EORTC-QLQ-BN20 questionnaire.

**Results:**

From October 2013 to March 2015, 594 patients were admitted for elective cranial neurosurgical procedure. 489 neurosurgical patients were screened for increased distress. Data from 450 patients could be correlated with the EORTC-QLQ-C30 and EORTC-QLQ-BN20. In 265 patients screening revealed increased distress. A concurrent reduced global health /higher rates of future uncertainty and conspicuous distress screening results are found in 173 patients (69.5%) compared to 30.5% of patients (n= 76) with unremarkable screening. Increased distress screening was highly significant with increased level of future uncertainty as well as decreased level of quality of life and global health (p<0.0001).

**Conclusion:**

Psychooncological distress is accompanied by reduced quality of life, global heath and increased future uncertainty. Therefore HQOL assessment can be helpful identifying patients with increased distress.

## INTRODUCTION

Patients with intracranial tumors are at high risk for developing affective disorders such as depression or anxiety [1, 2]. A prevalence of depressive symptoms in 15-38% in these patients is reported [3, 4]. Early diagnosis and treatment of psychological distress is of high relevance to hamper the handicap of comorbid psychological disorders and facilitate an optimized medical treatment [5]. Therefore, early identification of patients with increased distress should be an important neurooncological treatment purpose. Especially, in the framework of a neurosurgical department where patients are confronted with diagnosis, upcoming inevitable therapies and prognosis of malignant diseases.

But although diagnosis of distress can be improved by screening with self-report questionnaires [6], routine screening assessments during hospitalization is not often implemented, especially not in the daily routine- neurosurgical setting mostly due to time consuming instruments and missing qualified staff. However, the European Organization for Research and Treatment of Cancer Quality of Life score questionnaire and European Organization for Research and Treatment of Cancer Quality of Life Questionnaire-Brain Neoplasm module questionnaires (EORTC-QLQ-C30 and EORTC-QLQ-BN20) questionnaire is more widely used and often included as quality of life assessment instrument in clinical neurooncological studies [7]. Therefore, we were interested if there is a clear coincidence between psychooncological distress and health related quality of life (HRQOL), hypothesizing that increased distress is reflected in the HRQOL assessment.

On that account, in this retrospective, observational study we correlated psychooncological screening results with the HRQOL questionnaire in electively admitted neurosurgical patients.

## RESULTS

### Patients

Since October 2013 till March 2015, 594 patients were admitted for an elective cranial neurosurgical procedure. 489 patients (82.32%) could be screened for their postoperative psychooncological treatment demand in this prospective observational cohort study. Because of missing or incomplete data 39 patients had to be excluded. Finally data of 450 patients could be further analysed. In 229 (50.8%) patients a malignant cerebral lesion was diagnosed (anaplastic astrocytoma WHO III (n=45), glioblastoma WHO IV (n=104), cerebral metastasis (n=63), cerebral lymphoma (n=17)). In 221 patients (49.2%) benign cerebral lesions as meningioma (n=106), pituitary tumors (n=35), vascular lesions (n=38) and trigeminal neuralgia (n=42) were diagnosed.

Perioperative neurological assessment via Karnofsky performance scale (KPS) revealed stable results with median KPS 90 (range 90-100) pre and postoperatively.

Patients’ characteristics are detailed in Table 1.

**Table 1:** Patients characterization and demographic data

	All patients (n=450)	Psychooncological assessment (n=265)	EORTC QLQ-C30-BN20 questionnaire (n=263)
	n/(%)	n/ (%)	n/ (%)
**Diagnosis**			
**malignant lesion**	229 (50.8)	145 (54.7)	147 (55.9)
anapl. glioma/GBM	149		
cer. metastaes	63		
cer. lymphoma	17		
**benign lesion**	221 (49.1)	120 (45.3)	116 (44.1)
vascular/trigeminal neuralgia	80		
pituitary tumor	35		
meningeoma	106		
**prim. diagnosis**	332 (73.7)	229 (86.4)	224 (85.2)
**recurrent disease**	128 (28.4)	36 (13.6)	39 (14.8)
**Age (years) median**	51.5		
<65 years	313 (69.5)	183 (69.1)	183 (69.6)
>65 years	137 (30.4)	82 (30.9)	80 (30.4)
**male**	213 (47.3)	118 (44.5)	115 (43.7)
**female**	237 (52.6)	147 (55.5)	148 (56.3)
**partnership**			
**yes**	359 (79.7)	202 (76.2)	205 (77.9)
**no**	91 (20.2)	63 (23.8)	58 (22.1)
**children**			
**yes**	324 (72)	191 (72.1)	191 (76.6)
**no**	126 (28)	74 (27.9)	72 (27.4)
**Pre-existing psychiatric disorders**			
**yes**	93 (20.6)	67 (25.3)	66 (25.1)
**no**	357 (79.3)	198 (74.7)	197 (74.9)
**ataractics**			
**yes**	86 (19.1)	67 (25.3)	66 (25.1)
**no**	364 (80.8)	198 (74.7)	197 (74.9)

2^nd^ column: data of patients with conspicuous findings in at least one distress screening instrument (HADS, DT, Po-Bado) and 3^rd^ column: data of patients with noticeable results in at least one of EORTC QLQ-C30-BN20 subscales (QoL, global health, future uncertainty).

### Psychooncological distress assessment

Psychooncological screening assessment was conspicuous in at least one questionnaire in 249 patients (55%) (n=132 with malignant disease, n=117 with benign disease, 110 male, 139 female, median age 54 years). Detailed screening results are demonstrated in Table 1.

Independent from a suspicious distress screening, patients were asked if further psychooncological support was required. 77 patients (17.1%) accepted and asked for further psychooncological assistance.

Regarding the different screening assessments 197 patients (43.8 %) scored a conspicuous (>6) screening result using the DT (median 5, mean 5.18). Using the HADS in 63 patients (14%) a high scoring (>=11) reflected increased distress (HADS-A= 49, HADS-D= 35 positive screening results). 126 patients (28%) demonstrated a striking screening result (scoring >=8) using the PoBado questionnaire. Patients taking ataractics demonstrated significant higher distress compared to patients without medication using the HADS (p=0.001) or PoBado (p=0.0001) screening assessments.

A positive screening result in one of these questionnaire was highly significant with increased level of future uncertainty as well as decreased level of quality of life and global health determined with the EORTC QLQ-C30-BN20 questionnaire (p<0.0001).

### HRQOL assessment

211 patients (46.8) presented reduced quality of life, 225 patients (50%) reduced global health status and 99 patients (22%) demonstrated a high distress concerning their own future. Here, especially patients with recurrent disease (p=0.016) demonstrated significant increase of their future uncertainty. Ataractic medication was reflected in a significant decrease of QoL (p=0.01) and global health (p=0.022). QoL was also decreased in patients with pre-existing psychiatric disorders (p=0.053). Detailed screening results are summarized in Table 2.

**Table 2:** Detailed illustration of the EORTC subscales future uncertainty (FU), global health (GH) and quality of life (QoL) including significant findings

	QoL (n=229)	p- Value	GH (n=217)	p-Value	FU (n=96)	p- Value
	n/ (%)		n/ (%)		n/ (%)	
**Diagnosis**						
malignant	127(55.5)	0,533	116 (53.5)	0,774	65 (67.7)	**0,003**
benign	102 (44.5)		101 (46.5)		31 (32.3)	
**prim. diagnosis**	160(69.9)	0,907	152 (70)	0,983	63 (65.6)	0,337
**recurrent disease**	69 (30.1)		65 (30)		33 (34.4)	
**Age (years) median**						
<65 years	161(70.3)	0,736	156 (71.9)	0,244	71 (74)	0,328
>65 years	68 (29.7)		61 (28.1)		25 (26)	
**male**	100 (43.7)	**0,018**	96 (44.2)	**0,022**	33 (34.4)	**0,001**
**female**	129 (56.3)		121 (55.7)		63 (65.6)	
**partnership**						
yes	177 (77.3)	0,782	171 (78.8)	0,679	72 (75)	0,678
no	52 (22.7)		46 (21.2)		24 (25)	
**children**						
yes	161 (70.3)	0,809	154 (71)	0,923	74 (77.1)	0,199
no	68 (29.7)		63 (29)		22 (22.9)	
**Pre-existing psychiatric disorders**						
yes	61 (26.6)	0,132	59 (27.2)	**0,025**	33 (34.4)	**0,006**
no	168 (73.4)		158 (72.8)		63 (65.6)	
**ataractics**						
yes	62 (27.1)	**0,001**	60 (27.6)	**0,002**	32 (33.3)	**0,001**
no	167 (72.9)		157 (72.4)		64 (66.7)	

### Comparison of HRQOL assessment and distress screening

**Figure 1:**
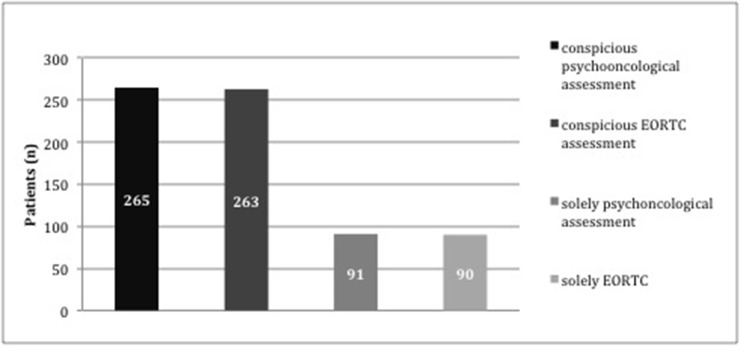
Block diagram- illustration of patients with conspicuous findings in one of the distress tools independent from the QoL questionnaire (n=265), patients with conspicuous findings in at least one of the QoL questionnaire subscales independent from the psychooncological distress screening results (n=263), patients with noticeable findings solely in at least one distress screening instrument but unremarkable QoL questionnaire (n=91) and patients with striking results in at least one subscale of the QoL questionnaire but unremarkable results in the distress screening (n=90)

In most patients quality of life assessment as well as distress screening were striking. 265 patients (58.8%) presented conspicuous distress screening results and decreased HRQOL at the same time. 263 patients (58.4%) declared decreased quality of life combined with increased distress. Only 91 patients (50.3%) and 90 patients (49.7%) demonstrated positive screening results in one assessment (psychooncological screening, EORTC questionnaire, respectively) (Figure 1).

**Figure 2:**
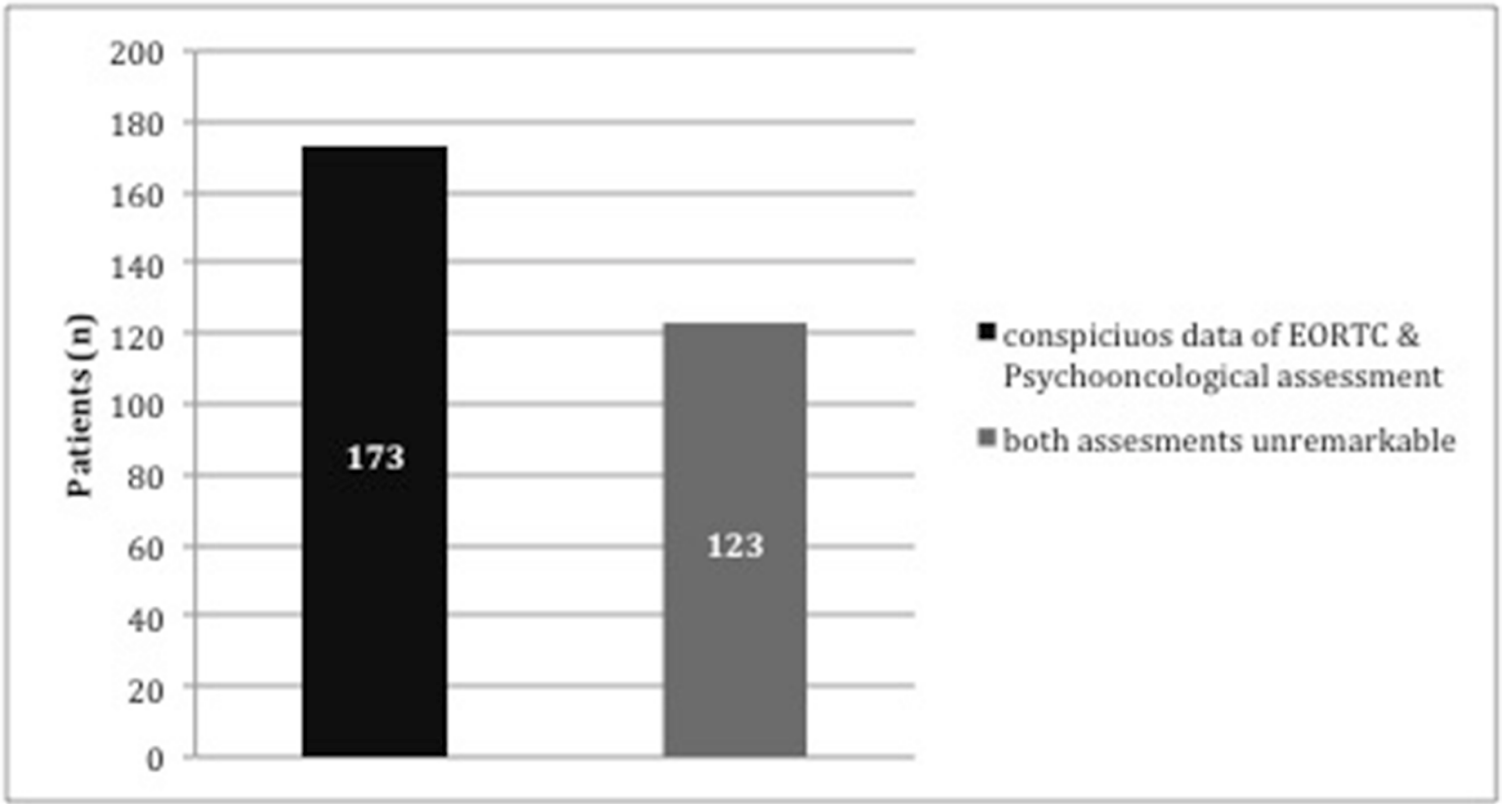
Block diagram- illustration of patients with conspicuous findings in both assessments and all subscales (distress and EORTC QLQ-C30-BN20 questionnaire) (n=173) vs. patients with complete unremarkable screening results (n=123)

To identify important sociodemographic data with impact on suspicious screening we compared patients with striking results in all aspects of both assessments with patients without conspicuous findings in any questionnaire (Figure 2).

Patients without any conspicuous findings were significantly male (p=0.03), patients without any psychological treatment prior to the neurosurgical diagnosis (p=0.014) and patients without ataractics (p=0.02). Interestingly diagnosis, tumor recurrence or sociodemographic factors were not significantly correlated to increased distress. Detailed results are summarized in Table 3.

**Table 3:** Demographic data and significant results of patients with conspicuous findings in both assessments and all subscales (distress and EORTC QLQ-C30-BN20 questionnaire) (1^st^ column) vs. patients with unremarkable screening results (2^nd^ column)

	QoL and distress conspicuous (n=173)	QoL and distress inconspicuous (n=123)	p-Value
	n/ (%)	n/ (%)	
**Diagnosis**			
malignant	98 (56.6)	64 (52.0)	0.425
benign	75 (43.4)	59 (48.0)	
**prim. diagnosis**	148 (85.5)	102 (82.9)	0.893
**recurrent disease**	25 (14.6)	21 (17.1)	0.310
**Age (years) median**			
<65 years	124 (71.7)	96 (78.0)	0.206
>65 years	49 (28.3)	27 (22.0)	
**male**	68 (39.3)	70 (56.9)	**0.003**
**female**	105 (28.3)	53 (43.1)	
**partnership**			
yes	135 (78.0)	70 (56.9)	0.448
no	38 (22.0)	53 (43.1)	
**children**			0.406
yes	125 (72.3)	84 (68.3)	
no	48 (27.7)	39 (31.7)	
**Pre-existing psychiatric disorders**			
yes	49 (28.3)	78 (14.6)	**0.014**
no	124 (71.7)	105 (85.4)	
**ataractics**			
yes	55 (31.8)	22 (17.9)	**0.02**
no	124 (68.2)	101 (82.1)	

## DISCUSSION

In this analysis, we correlated postoperative psychooncological distress screening and HRQOL assessments of 450 neurosurgical patients, presenting - to our knowledge- the largest series in literature.

Recent publications clearly demonstrated the clinical impact of increased psychooncological distress in neurosurgical patients [3, 8, 9, 10] and could illustrate a correlation between elevated distress and patient compliance during further therapy [3, 11]. But still standard psychooncological assessment tools do not exist, particularly not in the daily routine of most neurosurgical departments.

In contrast health related quality of life (HRQOL) assessment has gained more importance in the last years not only in the framework of clinical trials as primary endpoint and outcome measurement [7]. Data about HRQOL in brain tumor patients are widely available demonstrating its impact as an independent predictor of therapy compliance and survival [12, 13]. Due to its ubiquity compared to the psychooncological screening for neurosurgical patients we were interested if HRQOL screening results assessed by the EORTC QLQ-C30/BN 20 could also reflect elevated distress during hospitalization in electively admitted neurosurgical patients.

Therefore we analyzed HRQOL assessment and distress screening results of electively admitted neurosurgical patients. The EORTC-QLQ-C30/ EORTC-QLQ-BN 20 questionnaires encompass diverse functional scales, symptom scales, single item scores as well as global health status, future uncertainty and global QOL, which is designed and validated for brain tumor patients [14]. Here, we concentrated on striking results regarding superordinate aspects of the questionnaire like global health, future uncertainty and QOL based on the following considerations: (1) to keep the evaluation as simply and suitable for daily use as possible (1) because of our inclusion criteria next to brain tumors our patients comprised different neurosurgical diagnoses, therefore we neglected brain tumor specific aspects (2) at the time of assessment our patients presented a median KPS of 90 without major neurologic deficits, thus we did not pay attention toward special functional and symptom scales. Additionally, a recent analysis highlighted future uncertainty and global health status especially in brain tumor patients as very important and correlated with unmet needs in these patients [15].

In literature there are only few data about analyzing distress and quality of life in neurosurgical patients. Kvale et al included 50 glioblastoma patients using the distress thermometer and the Functional Assessment of Cancer Therapy- Brain (FACT-Br) questionnaire and reported a significant correlation between the distress score and the social well being as well as the emotional well being subscales of the FACT-Br questionnaire [5]. In a larger multicenter study Hickmann et al presented data of 167 neurooncological outpatients analyzing results of the distress thermometer, the EORTC QLQ-C30/BN 20 as well as the Supportive- Care-Needs- Survey- SF34-G (SCNS). They observed a strong correlation of elevated distress and fatigue, cognitive- emotional function, global health status and future uncertainty [15].

In 265 patients (58.8%) and 263 patients (58.4%) increased distress and conspicuous HRQOL findings were observed. Dependent from the different distress- screening tool in this early postoperative screened patient population 14-48% increased distress was diagnosed. This range is reflected in previous analyses, where the prevalence of depression in brain tumor patients varies from 15 to 38% [3]. Elevated distress is observed in 28-52% patients with intracranial tumors [5, 16].

The effect of the distress screening time point (preoperatively- postoperatively- during course of therapy) on the screening results remains unclear [17, 18, 19]. A preoperatively increased distress is observed due to the fear of surgical intervention or the risk of death, coma or neurological and physical deficits after surgery [9]. In a longitudinal distress analysis Ronney and collogues described a stable gradient during the course of therapy [20]. Here, we concentrated on the early postoperative time- further longitudinal analysis are in progress. Independent from their distress screening results further psychooncological assistance was offered to all patients. Interestingly only 77 patients (17.1%) accepted this subliminal offer, which may be explained by the screening time point. This aspect will be further observed by an upcoming longitudinal analysis of our data.

The impact of medical data, diagnosis or sociodemographic data on increased distress remains unclear and is discussed controversially in literature [9, 11, 21, 22]. In this analysis the distribution regarding diagnosis, age, sex, social and medical background in patients with increased distress and patients with decreased QoL are comparable (Table 1), demonstrating no significant impact of diagnosis, tumor recurrence or sociodemographic data on distress or QoL. To further elucidate possible impact factors we compared patients who presented increased distress as well as decreased QoL in all analysed aspects with patients who did not present any remarkable screening assessment (Figure 2, Table 3). Patients without any conspicuous findings were male (p=0.03), without any psychological treatment prior to the neurosurgical diagnosis (p=0.014) and without ataractics (p=0.02). The predominance of female patients with increased distress may refer to the large amount of meningioma patients in our patient cohort, where an association with postoperative psychological sequela is known [23, 24, 25]. In a large prospective cohort study Rooney and colleagues analysed psychooncological distress in the longitudinal illness course and could identify functional impairment and concurrent major depressive disorders as independent associated factors with increased distress [20], supporting our results.

Most important our analysis could demonstrate an accordance of increased distress and conspicuous findings regarding global health, future uncertainty and Quality of life assessed by the EORTC QLQ-C30/BN 20 questionnaire. Concurrent results were found in 69.5% of our patient cohort. A positive distress screening result was highly significant with increased level of future uncertainty as well as decreased level of quality of life and global health determined with the EORTC QLQ-C30-BN20 questionnaire (p<0.0001). Therefore, we can conclude, that increased psychooncological distress can be reflected in the QoL questionnaire.

Limitations of this analysis are: (1) the retrospective study design. (2) Our results reflect the distress situation only at one time point during hospitalization postoperatively, therefore these results need to be evaluated in a longitudinal study design. (3) Because there are no standard screening tools to determine the psychooncological distress of neurosurgical patients, we used three different tools demonstrating increased distress between 14-48% of all patients. In a larger multicenter study we try to identify a suitable screening instrument for neurosurgical patients. (4) Because of our inclusion criteria and to keep the comparison as simple and clearly as possible for the neurosurgical daily use, we concentrated on specific subscales of the EORTC QLQ-C30-BN20, therefore our results did not reflect the complete questionnaire.

Prompt identification of increased distress is becoming a major concern in multimodal neurooncological treatment. Several studies could clearly demonstrate the impact of distress on patient compliance and in conclusion on survival [12, 26, 27]. Therefore, distress screening should be implemented in the neurosurgical daily routine. If this is not available, our data highlight that the EORTC QLQ-C30-BN20 questionnaire could help to identify patients with increased distress.

## MATERIALS AND METHODS

### Patients

Inclusion criteria for this retrospective, single-center study were (1) diagnosis of an intracranial lesion, (2) elective admission for a neurosurgical procedure, (3) postoperatively assessment of all three questionnaires, (4) age > 18 years and (5) confirmed written consent. Exclusion criteria were palliative care and physically or cognitively disability to complete the questionnaires. Independent from their screening results patients were asked, whether a psychooncological consultation was requested.

The study was approved by the local ethic committee (number 4087).

### Psychooncological screening assessment

Screening was performed postoperatively (median 2 days post OP, range 1-4 days). At screening time point patients were informed about diagnosis, extent of resection and if further adjuvant treatment (radio-, chemotherapy or both) was needed. Standard postoperative medication included low dose steroids. The following neurophysiological tests were used:

#### Distress thermometer (DT)

Developed by the National Comprehensive Cancer Network (NCCN) the DT is a single-item 10 point visual analogue scale, which measures psychological distress. It consists of a scale from 0-10 (“no distress” to “extreme distress”) and a problem list comprising of 40 items representing commonly experienced problems. In brain tumor patients a score greater than or equal to 6 is recommended as in indicator that a patient is distressed and needs support [28].

#### Hospital anxiety and depression scale (HADS)

The HADS is a questionnaire widely used to assess symptoms of anxiety and depression in patients with somatic complaints, with excellent reliability and validity. It consists of 14 items, with seven per subscale. Items are scored 0, 1, 2, or 3, which gives a range of scores from 0 to 21 for each subscale. Two recommended thresholds are described: greater or equal to 8 (for greater sensitivity) and greater or equal to 11 (for greater specificity). Patients scoring ≥ 11 suffer from probable anxiety or depressive disorders [8, 28].

#### Psycho-oncological base documentation (PO-Bado)

Questionnaires may be difficult to fill out by neurooncologic patients themselves because of a lack of understanding or neurologic deficits. Therefore we were interested if an external assessment tool may be more reliable than self-administered assessment tools. To our knowledge since now there is no study analyzing the impact of the Po-Bado for brain tumor patients. Developed by the DAPO and PSO (German Associations for Psychooncology), the PO-Bado is developed as an external assessment tool. In contrast to the other questionnaires, the PO-Bado is filled out by the physician and not by the patient to estimate the patients’ subjective distress in the last three days in about 20 to 30 minutes. The questionnaire consists of six items scoring from 1-4. Scoring of 8+ is equalized with elevated psychooncological distress.

### Health related quality of life (HRQOL) assessment

#### EORTC-QLQ-C30 and EORTC-QLQ-BN20

The internal assessment tool for brain and neck cancer patients was developed by the European Organization for Research and Treatment of Cancer (EORTC) to evaluate the health related quality of life of cancer patients. It consists of 48 items scoring from 1-4 (“not at all”, “a little”, “quite a bit”, “very much”) and two items (“global health” and “quality of life”), which are scored from 1-7 (“very bad” to “excellent”). A high score in one of these items illustrates a high health status or a high QOL level. The EORTC QLQ-C30 comprises five function scales (physical, role, emotional, cognitive and social), three symptoms scales (fatigue, nausea/vomiting and pain) six single items (dyspnea, insomnia, appetite loss, constipation, diarrhea and financial effect) and global health. The Brain Cancer Module (BCM20) consists of 20 tumor specific items that assesses visual disorders, motor dysfunction, communication deficit, specific disease symptoms and future uncertainty. The EORTC QLQ-C30 and the BCM20 were scaled and scored after the recommended scoring manual of the EORTC.

Here, we neglected single item and function scales results because (1) we included all neurosurgical patients with a planned cranial surgical procedure independent from the diagnosis and (2) in all patients screened postoperative no major neurological symptoms were observed. A recent analysis highlighted future uncertainty and global health status especially in brain tumor patients as very important and correlated with unmet needs in these patients [15]. Therefore, our emphasis in this setting was to analyze noticeable results of future uncertainty (scoring > 2.75), global health (scoring <4) and quality of life (scoring <4) in correlation to striking results of DT, HADS and Po Bado questionnaires.

### Data collection

Epidemiological data, data regarding tumor location and histopathological appearance were collected from charts and electronic records.

A malignant lesion was defined as WHO° III/IV tumor, cerebral lymphoma or a cerebral metastases and a benign lesion as meningioma, pituitary tumor, vascular lesion or neuralgia.

### Statistical analysis

Statistical analyses were performed with GraphPad Prism version 5.00 for Windows and IBM Statistical Package for Social Sciences (SPSS) 24.0. Variances between independent groups were examined with Mann- Whitney- U Test in Prism (Prism5 for Windows; Version 5.01, GraphPad Software, Inc). Frequency of distribution was further analyzed with Chi^2^- Test performed with IBM SPSS 24. Before using non- parametric tests we were considered data for normal distribution (Kolmogorov- Smirnov- test).

In all test a significance was considerers when p <0.05.

## CONCLUSION

Independent from diagnosis increased distress is of high prevalence in neurosurgical patients. If screening assessments are not available, the EORTC QLQ-C30-BN20 subscales future uncertainty, global health and quality of life are good predictors for distress.
